# Event-related potentials evoked by passive visuospatial perception in rats and humans reveal common denominators in information processing

**DOI:** 10.1007/s00429-019-01854-4

**Published:** 2019-03-11

**Authors:** M. F. A. Hauser, V. Wiescholleck, J. Colitti-Klausnitzer, C. Bellebaum, Denise Manahan-Vaughan

**Affiliations:** 1 Department of Neurophysiology, Medical Faculty, Ruhr University Bochum, Universitaetsstr. 150, MA 4/150, 44780 Bochum, Germany; 2 International Graduate School of Neuroscience, Bochum, Germany; 3 Institute for Experimental Psychology, Heinrich-Heine University of Düsseldorf, Düsseldorf, Germany

**Keywords:** Passive visual perception, Rodent, Human, Event-related potential, Spatial information, Electrophysiology

## Abstract

In the human cortex, event-related potentials (ERPs) are triggered in response to sensory, cognitive or motor stimuli. Due to the inherent difficulties of conducting invasive mechanistic studies in human subjects, little is known as to the precise neurophysiological mechanisms that lead to their manifestation. By contrast, although much is known about synaptic and neural mechanisms that underlie information processing in rodents, very few studies have addressed to what extent ERPs are comparable in rodents and humans. Here, we explored this by triggering ERPs in both species during the passive observation of visuospatial imagery, shown in an oddball-like manner, using an experimental design that was equivalent. Several ERP-components were identified in the rodent cohort, corresponding, for example, to the human P1, N1, and P2. ERPs that are likely to reflect a rodent N2 and P300 were also detected. Deviance, as well as repetition effects were evident in both species, whereby rodent ERPs displayed more immediate response alterations to repeated stimuli and humans showed more gradual response shifts. These results indicate that humans and rodents may implement similar strategies for the passive perception and initial processing of visuospatial imagery, despite clear differences in their sensory and cognitive capacities.

## Introduction

The pathways supporting the perception of objects in humans and non-human primates have been the subject of many studies for almost half a century. The ventral stream, extending from V1 to V2 and V4, and the inferotemporal cortices (Ungerleider and Mishkin 1982; Mishkin et al. 1983; Felleman and Van Essen 1991) have been suggested to support the processing of objects in particular. Within area V1 of the primary visual cortex, neurons exhibit tuning towards basic visual features, such as luminance and spatial frequency, that are then combined along V2 and V4 to generate increasingly complex representations (Gallant et al. 1993; Ito and Komatsu 2004). Finally, in the inferotemporal areas, complex object categories, such as body parts, faces, and inanimate objects are represented (Perrett et al. 1982). Along this pathway, neurons thus show responses to increasingly complex feature combinations, while at the same time also showing increasing receptive field sizes (Gross et al. 1969).

Although much of our knowledge with regard to object perception comes from primate research, a recent increase in interest in rodent object perception now sets the stage to consolidate findings from mechanistic studies that feature rats and mice as the dominant species, with studies on higher cognitive functions conducted in humans and non-human primates. Studying the underlying neuronal response properties, several studies have shown that along the trajectory from V1, through latero-medial, latero-intermediate, latero-lateral, and occipito-temporal areas, an increasing tolerance to position and viewpoint is observed in the awake rat, similar to what is seen in primates (DiCarlo et al. 2012; Vermaercke et al. 2014; Tafazoli et al. 2017). Furthermore, it has been shown that V1 displays the shortest response latencies at around 40–50 ms, closely followed by latero-medial, and -intermediate areas at around 50 ms, after which latero-lateral and occipito-temporal areas respond at roughly 80 ms (Vermaercke et al. 2014). However, while neurons in the rat primary visual cortex exhibit tuning similar to that observed in primate V1, they lack functional columns (Ohki et al. 2005). Despite this difference, it was reported that, when rodents process multiple stimuli, neurons along the axis of tested regions become sharply energy-independent, meaning that luminance of presented objects had less of an impact in higher order areas (Tafazoli et al. 2017). The same study demonstrated that neurons in higher visual areas become increasingly view-invariant to objects. These findings suggest, that at least on a single-unit level, a similar progression of information processing along the visual areas occurs in rats, humans and non-human primates. To what extent the temporal dynamics of cortex-wide responses are comparable between rodents and primates is, however, not clear.

In this study, we set about to explore to what extent passively viewed visuospatial information is processed in equivalent ways by the rodent and human cortex. To do this, we created a test paradigm that was designed to investigate cortical novelty responses in the context of object processing by means of event-related potentials (ERPs). We observed that humans and rodents appear to utilize similar strategies for the passive perception and initial processing of visuospatial imagery.

## Experimental procedures

**Fig. 1:**
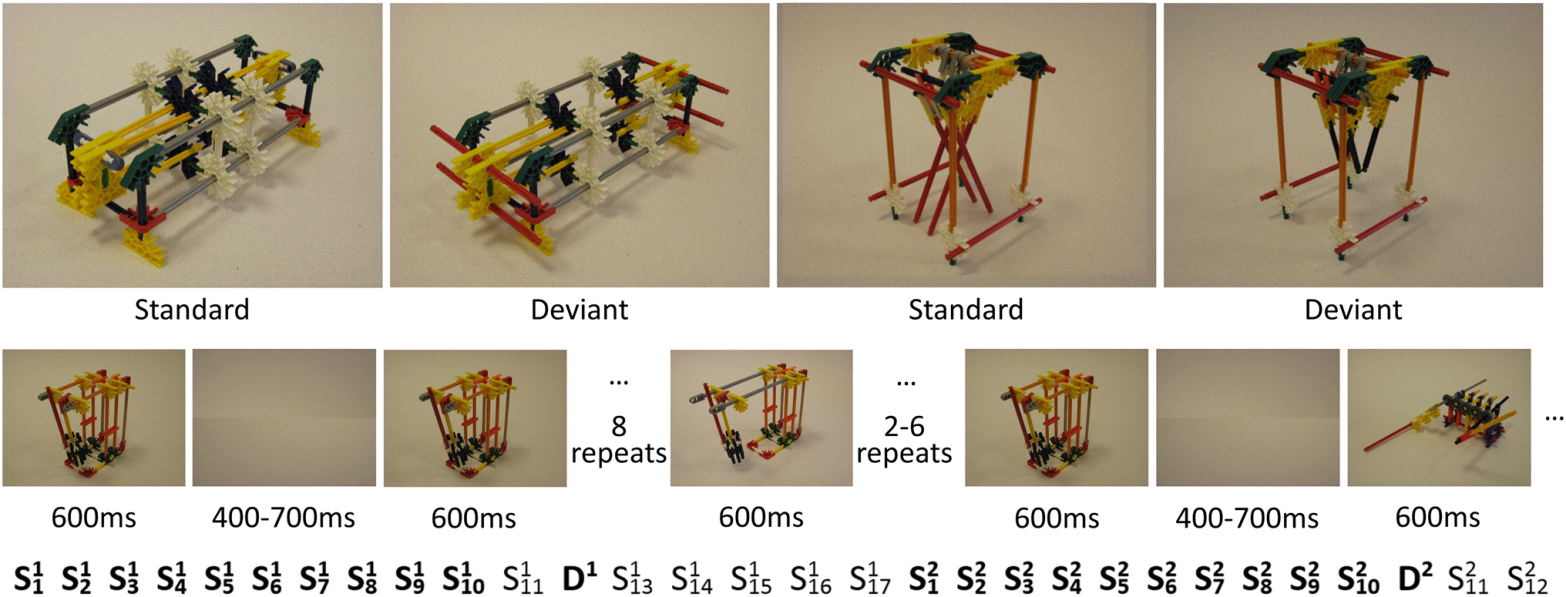
Stimuli, time-line and trial order. Top row: examples of the stimuli used. Middle row: timing of an example trial for the rodents. Bottom row: ordering of standards and deviants for two objects. The superscript denotes objects being shown as standard (S) or deviant (D). The subscript denotes presentation number. Bold letters indicate which epochs were later extracted for analysis

To compare evoked potentials in humans and rodents, we used a passive visuospatial oddball-like paradigm, where both species were presented with novel abstract complex objects in a repeated manner (standards), interleaved with the same objects in a configurationally changed variant (deviants; Fig. 1). In contrast to the classical oddball paradigm, where standards are repeated throughout the experiment, we changed the standard objects every 17–21 repetitions. The animals were shown these objects via two monitors while they were drinking from a juice dispenser (Kemp and Manahan-Vaughan 2012), during which we recorded EEG signals from 12 cranially implanted electrodes spanning from the frontal to occipital cortices. Our human subjects were exposed to the same paradigm whilst participating in a distractor task, to ensure they were looking at the objects, but not actively attending them.

### Rodent study

The rodent study was carried out in accordance with the European Communities Council Directive of 22 September 2010 (2010/63/EU) for care of laboratory animals. All experiments were performed according to the guidelines of the German Animal Protection Law and were approved by the North Rhine-Westphalia State Authority (Landesamt für Naturschutz, Umweltschutz und Verbraucherschutz, Nordrhein Westfalen, Germany). All efforts were made to reduce the number of animals used.

The animal cohort consisted of 13 adult male hooded Lister rats (Charles River Breeding Laboratories, Sulzfeld, Germany). At the time of surgery they were 7–9 weeks old. The animals were housed separately on a 12 h light/dark cycle, and under controlled temperature (22 ± 2 °C) and humidity (55 ± 5%). Both food and water were provided ad libitum.

### Electrode placement

To be able to differentiate ERP-components according to their cortex-related topographical properties, 13 stainless steel screws (with 0.8 mm diameter, a length of 1.4 mm, and an impedance of < 1 Ω) were implanted into the animal’s skull to enable recording of cortical EEG. Each screw (Optotec, Rathenow, Germany) was attached to silver wire (Axona, St. Albans, UK). Twelve screws served as recording electrodes and one was used as a ground electrode. Screw insertion was done while the animal was deeply anesthetized using sodium pentobarbital (Narcoren, Merial GmbH, Germany, 52 mg/kg, intraperitoneally). The animal was placed in a stereotactic unit. Following application of the local anesthetic, lidocaine, a scalp incision of ca. 3 cm in length was made from a point between the eyes, along the midline towards the back of the skull and the periosteum was then removed. Small drill holes were then made in the skull to correspond to the electrode positions. The drill holes were positioned in five rows that were aligned 3 mm on either side of the midline (see: Fig. 6h). Two rows were positioned 1.5 mm and 4.7 mm anterior to bregma. At these anterior positions two further electrodes were placed exactly on the midline. Three rows of two electrodes were placed 1.9 mm, 4.16 mm, and 6.9 mm posterior to bregma. The ground electrode was placed in the skull over the cerebellum. This arrangement of electrodes allowed covering the largest portion of the rodent cortex, while at the same time allowing some degree of specificity to different functional areas. The electrode screws were inserted without piercing the dura. After placement the screws were then attached via the silver wire to a 16-channel Headstage (Axona, St. Albans, UK). The connections were covered and the assembly was attached to the skull using dental acrylic (Paladur, Heraeus Kulzer, Wehrheim, Germany). Pre-and postoperative analgesia was implemented using Meloxicam (Boehringer Ingelheim Vetmedica GmbH, Ingelheim, Germany). At least 10 days were allowed to elapse after surgery before apparatus training and experiments were commenced.

The most anterior row of electrodes covered the frontal association cortices (FrA and FrAz) (see: Fig. 6h). The second row of anterior electrodes covered the primary motor cortex (M1) laterally, and on the midline the cingulate cortex area 1 (Cg1). The third row (immediately posterior to bregma) covered the primary somatosensory areas (S1) (see: Fig. 6). The fourth row covered the parietal association cortices (PtA), whilst the last row was situated above the primary visual cortex (V1M).

### Recording apparatus

To ensure that the rodents viewed the stimuli presented, we implemented a similar approach as already described in a previous study (Kemp and Manahan-Vaughan 2012): animals were placed in a recording chamber made of gray Perspex that contained a triangular annex where, through a hole (6 cm above ground), the animals could drink from a nozzle of a drinking bottle filled with carrot juice mixed with honey. The two flanks of this triangle-shaped part of the box contained removable windows made of non-reflective clear Perspex. Two monitors were placed behind these windows, one on each side, at a distance of 40 cm. Apart from the roof of the box, which was open to allow free movement of the cable connecting the headstage to the amplifier, the outsides and lower side of the box were covered in tin foil, which was grounded via a cable attached to the input connection of an electrophysiological recording system (Axona, St. Albans, UK).

To familiarize them with the recording procedure, the animals were handled individually for 10 min per day for at least a week before the start of the experiment. A key element of the experimental design is that the animals choose a position at the windows of the recording chambers so that they can view both computer monitors simultaneously. To achieve this, during the experiment they were able to drink carrot juice in this position. For at least 3 days before the experiment, the animals spent around 30 min per day in the recording chamber, during which they were able to drink the juice ad libitum. During this time, the monitors, on which images were later displayed, were turned on, to habituate the animals to the level of luminance coming from the screens.

### Stimuli and experimental trials

A randomized passive visuospatial oddball-like paradigm was designed to investigate the effects of stimulus repetition, as well as spatial change, on ERP-components. The stimuli used in this set-up were the same as those employed by Bellebaum et al. (2013; Rüther et al. 2014a, b; Ghio et al. 2016) (Fig. 1). These images consisted of photos of 15 abstract objects that were constructed from toy construction parts (K’NEX™). These stimuli were selected for the present study, as they had been created and standardized such that they were complex and real, yet provided little to no association with any meaningful objects, and, therefore, eliminated any affordance humans might perceive in them. This effectively allowed presenting our human subjects with images that were as novel to them as they were to the rodent cohort. The objects were photographed so that the three-dimensional configuration of the structure was easily visible (Fig. 1). For each object, two corresponding deviants were constructed, in which the standard object was slightly altered. These alterations consisted of replacing a certain component of the object, effectively changing its spatial configuration (Fig. 1). The sequence of events in one particular experimental trial of the experiment was as follows: for both, humans and rodents, one standard stimulus was repeatedly shown between 17 and 21 times, after which a new standard was introduced. For one of these repetitions the deviant was shown instead of the standard, randomly from the 11th to the 15th repetition. The initial presentation of a standard, and its corresponding ERP is what we here refer to as the novel object, and the corresponding novelty response, whilst the configurational change in the deviant stimulus elicited the deviant response.

### Procedure

For the experiment, the animals were placed in the recording chamber. Throughout the experiment, the room lights were turned off. Each image was presented for 600 ms, with a random inter-stimulus interval between 400 and 700 ms. This was held constant throughout each entire experimental block, thus no planned breaks or different intervals were implemented when a new standard, or the deviant was shown. Importantly, images were only presented while the animals were drinking to ensure that their heads were correctly placed to view both computer screens. Whenever they stopped, the presentation was paused manually by the experimenter, and only restarted after the animals resumed drinking. If at any point the animals stopped drinking for more than 5 s, all events up until the presentation of the next object were discarded from analysis. The rodent experiment was carried out over at least 4 days. On each day, the experiment was further divided into four sessions. Each session consisted of six trials in which the stimuli were presented as described above. Between the sessions, the drinking bottle was removed for around 3–5 min. In five animals, data acquisition was extended beyond four days (for three animals this meant a total of 5 days, for two animals 6 days), because the animals did not want to drink for more than 30 min in one session, so that the experiment was aborted.

### Data acquisition and preprocessing

During the recording sessions, aheadstage, serving as an AC-coupled unity-gain operational amplifier, was plugged onto the 16-channel connector, from where the signal was passed through the Axona data-acquisition system where it was amplified to be passed on to the recording computer. Finally, the continuous EEG data were exported to Matlab at a sampling rate of 250 Hz for further analysis. There, using the EEGLAB toolbox (Delorme and Makeig 2004), the data from each recording session were filtered using a high- and low-pass cut-off frequency of 0.5 and 70 Hz, respectively. After this, the individual channels were assessed and, if consistently noisy as evidenced by clear and broad departure from other electrodes in its power spectrum, removed from further analysis. In two animals, this was the case for the right frontal electrode, where the left counterpart was used to replace the missing electrode. The left somatosensory channel contained too many artifacts in eight of the 13 animals, thus, we chose to exclude it entirely from further analysis. As one of our main aims, next to exploring visuospatial deviance, was to investigate the effects of stimulus repetition on the processing of visual information in our two populations, we analyzed the first ten repetitions of every presented standard. These ten repetitions were the minimum number of presentations of the standard before a deviant could potentially occur. For these conditions of interest, the data were segmented into epochs of 1200 ms, starting from 200 ms before the presentation of a given image. The data were re-referenced to the average of all electrodes, after which, by means of visual inspection, noisy and unusable trials were removed. Specifically, we excluded epochs where the signal was partially clipped or lost, due to the juice coming into contact with the surrounding aluminum foil and the rodent at the same time. Independent component analysis, as implemented in the EEGLAB toolbox was used to decompose the data into as many temporally uncorrelated components as there were electrodes. This allowed subtracting noise and artifacts that were generated by the drinking motion, while having a relatively small impact on the signal of interest. In most cases this was just a single component, identified through being represented in the data during the baseline period, as well as the time after stimulus onset, and being equally prominent in all electrodes. Care was taken to minimize the impact this subtraction had on component amplitudes, while resulting in the smoothest possible baseline. Finally, after baseline subtraction, all epochs belonging to a particular condition were averaged per channel.

## Human study

A total of 30 healthy, medication-free, adult participants with normal or corrected-to-normal vision participated in this study, which received prior approval from the Ethics Committee of the medical faculty of the Ruhr University Bochum. After excluding three individuals due to too many trials (> 50%) being rejected by the automatic artifact rejection algorithms (see “Data acquisition and preprocessing” section), the final sample consisted of 16 females and 11 males, with a mean age of 30.5 years.

### Procedure

The participants were seated between 70 and 90 cm from the screen. The stimulation in each individual trial of the task used in human participants was the same as for the rodents (see above). To ensure that the human participants were only passively processing the objects, they were given a distractor task, which required the visual discrimination of a small square that appeared in the centre of the screen after the onset of the object picture. Participants responded with a right-or left-handed button press, depending on whether the square was white or black. On-screen instructions explained the task, and that they were to respond as fast as possible. Before the actual experiment started, a short training trial was presented. Participants were presented the images for at least 600 and maximally 800 ms before the target square appeared, lasting until a response was made, or until a total of 1200 ms since the appearance of the image had passed. As was the case for the rodents, the inter-stimulus interval was between 400 and 700 ms. Furthermore, the experiment consisted of four blocks, with each block consisting of 15 trials (one per object), and their respective deviants. The deviants alternated from one block to the next. Between these blocks, participants were able to take a break.

### Electrode placement

Using the ActiCap system (Brain Products GmbH, Munich, Germany), the participants were fitted with a 64-Ag/AgCl-electrode cap, which was secured with a chin-strap and configured according to the 10% extension of the international 10–20 system. All electrodes were referenced to FCz, with the ground located at AFz.

### Data acquisition and preprocessing

Before the recording, the electrodes were filled with electrode gel, and impedance was kept below 5 kΩ. The signal was digitized at 500 Hz. Using BrainVision Analyzer (BrainProducts GmbH, Munich, Germany), the data were ‘cleaned’ using independent component analysis to identify eye-blink artifacts, and subjected to a bandpass filter using 0.5 and 70 Hz as low and high cut-off frequencies, respectively. After that, as in the rodent preprocessing pipeline, the data were segmented into epochs of 1200 ms length, from 200 ms before to 1 s after an event of interest. Automatic artifact detection, with a maximum voltage step of 50 µV, and an epoch-wide maximal and minimal amplitude difference of 150 and 0.5 µV, respectively, was then applied to remove trials with artifacts.

## Analysis

### Source analysis

For both cohorts, we first performed a source reconstruction of the recorded ERPs over time, to allow identification of the different processing stages. Note that for the humans’ source reconstruction we used a subset of 14 individuals that showed the strongest source power in the occipital cortex during the P1 component (defined as greater than a 5% signal increase relative to the baseline). The source estimation was done using the Matlab-toolbox fieldtrip (Oostenveld et al. 2011). For the rat cohort, the forward model was built based on a rodent T1-scan published by Valdés-Hernández et al. (2011). Specifically, we created a mesh of the combined gray and white matter estimates, upon which we added a thin layer of a second mesh representing extracerebral fluid. We chose not to distinguish between white and gray matter in the forward model, since it was difficult to meet the requirement of the meshes being non-intersecting and closed, and because we were interested mostly in superior cortical, thus gray matter areas, that we deemed more likely to be estimated correctly with the low spatial resolution provided with our electrode number. A head model was then created using the openmeeg-software (Kybic et al. 2005; Gramfort et al. 2010). Volume conduction for the gray and fluid volumes was set to 0.33 and 1, respectively, and the two layers consisted of 3998 and 1998 vertices, respectively. The leadfield matrix was created based on this head model and on ten electrodes (excluding both electrodes above the somatosensory cortices), as well as a rectangular 29 by 28 by 7 source grid of 5684 nodes spaced 0.5 mm apart, spread across the neocortical region. Again, we chose to focus on the superior cortical areas due to our two-dimensional electrode placement, thus only created a depth of 7 (3.5 mm) layers. For the human cohort, we used the boundary element head model provided as a part of the fieldtrip toolbox, which consists of three layers, being scalp, skull, and brain. The human source-grid consisted of 15 by 18 by 15 sources spaced 1 cm apart.

We used linear constrained minimum variance, a beamformer scanning method, where each potential dipole is estimated separately. This method constructs a spatial filter to separate one source from the next, by creating the filter for each source such that when it is applied to another source, the resulting variance is as close to zero as possible. This is done using a covariance matrix derived from the data in question, which here were all epochs belonging to first and tenth presentations. We calculated the filter based on all time points in these epochs, thus from − 200 to 1000 ms. We used the tenth presentation as a baseline, to which the novelty response was compared. Subsequently, we used the filter to estimate the source power of the novelty response relative to the baseline across 40 ms time windows, centered on each time point starting from stimulus onset. Source power of the novelty response was calculated at each time point by subtracting the estimate for the baseline from the estimate for new objects, and dividing it again, by the estimate for the baseline (the percentage-wise difference).

### Multivariate analysis of source estimates

One important question was to what extent the human, and especially the rodent, source estimates could be used to identify different processing stages, that in turn could corroborate the choice of ERP-components to analyze. For this, we correlated the brain patterns elicited at every time point to all other time points. This yielded a matrix of *R* values indicating to what extent source estimates were coherent over time. Naturally, and in part due to the calculation of source estimates spanning over time windows of 40 and 80 ms in the rat and the human cohorts, time points adjacent to one another produced larger correlations than those further apart in time. However, we found some areas along this temporal axis produced broader correlations than others, which were indicative of less transient patterns, that may constitute a “processing stage”. To quantify this statistically, we employed permutation statistics, whereby we randomly permuted the source estimates along time points and correlated this with the original, non-permuted data a total of 1000 times. This effectively created a distribution of what we could expect if the time points were interchangeable (the null-hypothesis distribution). We then ‘thresholded’ the original matrix according to how many of the correlations with permuted data yielded stronger *R* values than the original. We selected a threshold of 50, corresponding to a *p* value of 0.05. To quantify different cortical states that contributed to the novelty response, and to relate these to the different components, we calculated a time series that indicated how stable the source estimates for a given time point were over time. This was done by counting the number of consecutive time points in which source estimates were significantly correlated, following each respective time point. Here, we allowed the ‘skipping’ of a single non-significant time point, if it was followed by further significant correlations. The resultant time series indicated different peaks, which coincided with many of the ERP-components for both rats and humans.

### Analysis of repetition and deviant effects

After identifying the different components of interest, we analyzed the effects of repetition and deviant effects separately in the electrodes in which we found the respective components to be the most prominent. Further analyses were done for each component using the subject-wise mean peak amplitude, for each of the first ten presentations, as well as the deviant. This amplitude was, in most cases (the exception being the human N2, and the rodent P3), the maximum/minimum of the condition mean in a time window of ± 30 ms for the humans, and ± 32 ms for the rodents, from the respective peak of the grand average component. This was chosen to account for subject-wise variability in component latency. To better control for type I errors, we averaged the last six repetitions into two conditions, consisting of repetitions 5–7, and 8–10 that we refer to as the intermediate, and late repetition phase trials, respectively. The repetition effects were tested using a one-way repeated measures analysis of variance (ANOVA) with the single factor of repetition number constituting six levels, from presentations one to four, as well as intermediate and late repetition phases.

If found to be significant, we then tested three functions in terms of their respective fit to each subject’s mean amplitudes over all ten repetitions. The first was a basic linear function, the second a quadratic, and the third a cubic polynomial. For these curves we evaluated the adjusted *R*^2^, which indicates the amount of variance explained by a curve, while penalizing (dividing by) the number of parameters. This was done separately in each subject, and the resultant *R*^2^_adj_ from the linear model was compared to both quadratic and cubic functions were compared in a Wilcoxon signed rank test to test whether the non-linear functions were significantly better at explaining the data. In cases where both, quadratic and cubic functions, were better at explaining the repetition effect, these were further contrasted. In the case of non-significance, the repetition effect was assumed to follow the more parsimonious model.

Finally, we conducted a separate analysis to investigate whether the deviant image was significantly different from the late repetition phase, using a paired *t* test, with *α* = 0.01 to account for multiple comparisons in each of the cohorts. For the rodent P3, there was no clearly discernible peak in the grand average, for which we, therefore, chose the condition-specific peak in a 300 ms time window, from 200 to 500 ms.

## Results

### Human novelty response progresses from V1 to inferior temporal cortex, and posterior midline

**Fig. 2:**
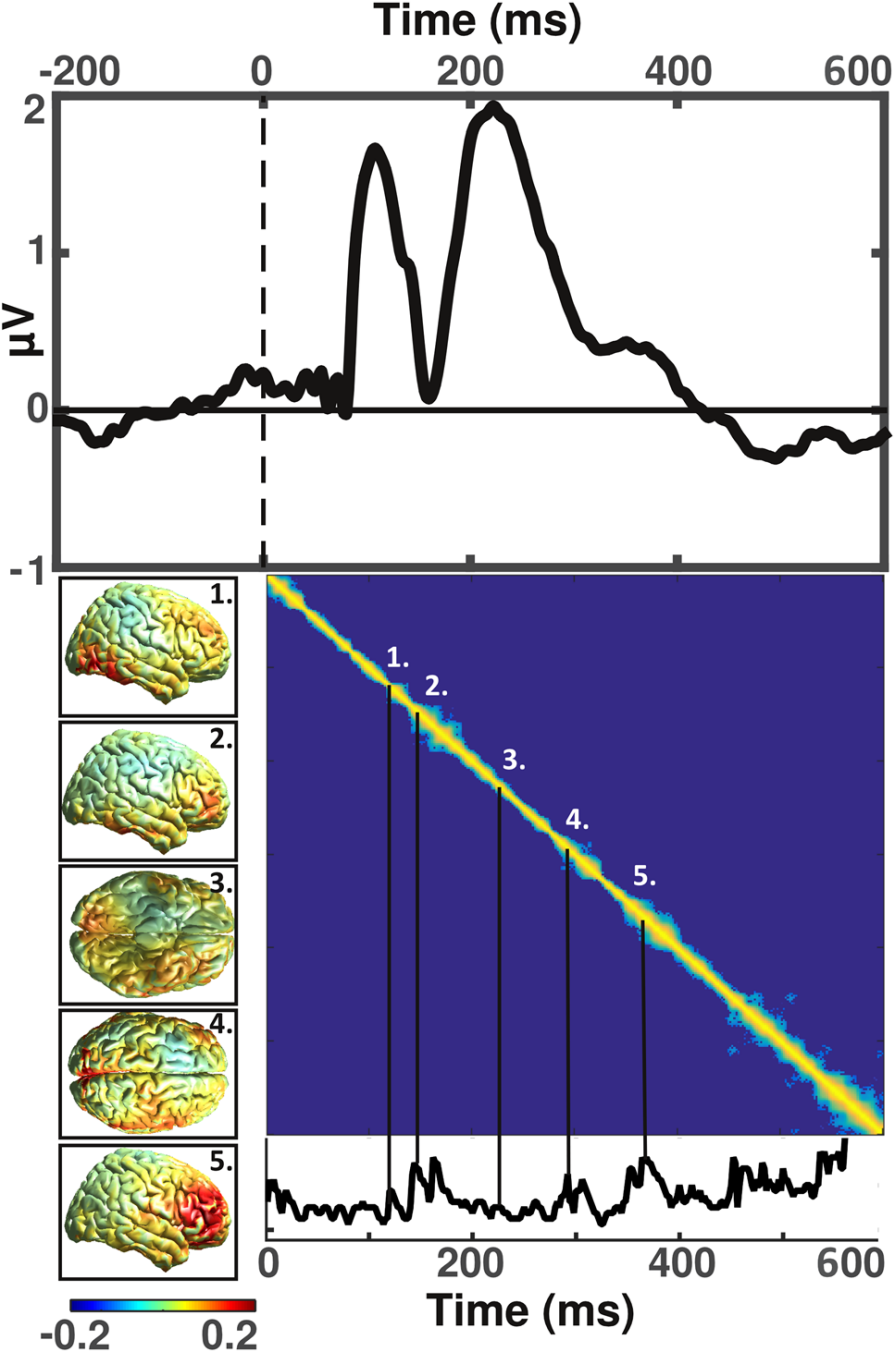
Event-related potential response and corresponding sources of human subjects. Top: human grand average event-related potential (ERP) derived from parietal electrodes. Bottom right: representational similarity matrix calculated for the time span of 0–600 ms and ‘thresholded’ at *p* < 0.05, that includes a time series indicating the time points at which source patterns correlated significantly. Bottom left: source estimates for time windows of interest corresponding to the numbering included in the bottom right panel

Closer scrutiny of the representational sample of our human cohort, on which we ran the source analysis, revealed a progression from visual cortices (Fig. 2, inset ‘1.’) to inferotemporal areas (Fig. 2, inset ‘2.’), and then a distributed activity pattern spanning temporal, parietal, and inferior occipital areas (Fig. 2, inset ‘3.’) that occurred in response to the presentation of novel visuospatial items (Fig. 1). Subsequently, the posterior midline region was found to be maximal (Fig. 2, inset ‘4.’), indicating a generation in the posterior cingulate. Finally, frontal activity was strongest (Fig. 2, inset ‘5.’). Particularly the occipital (at 120 ms), inferotemporal (144 ms), posterior medial (292 ms) and frontal (352 ms) increases in source power, coincided with an increase in temporal stability over time (Fig. 2, bottom).

### Human ERP-components reflect the different stages of the novelty response

**Fig. 3:**
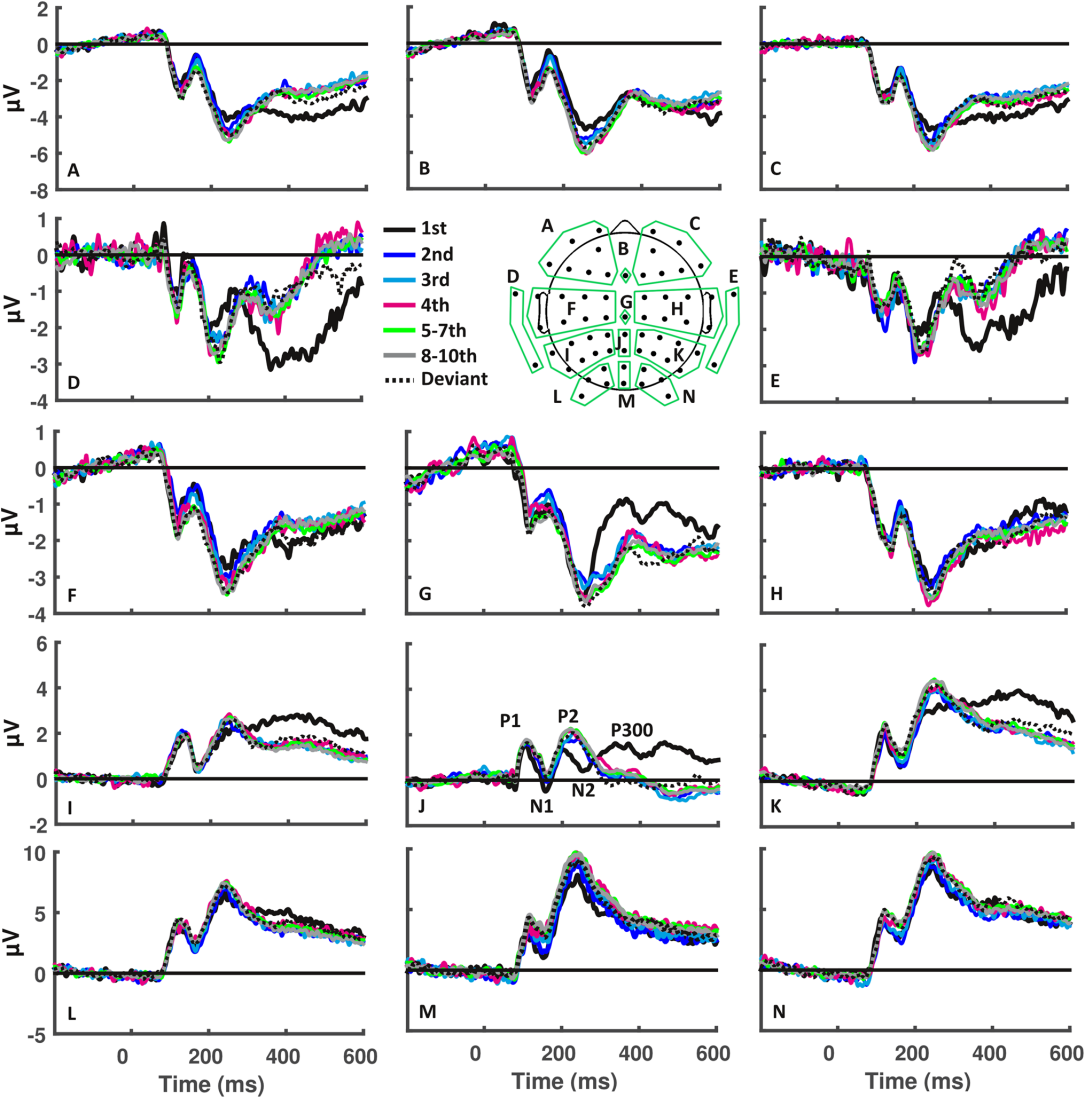
Human event-related potentials elicited by visuospatial information processing. Event-related potentials (ERPs) were recorded across 14 subsets of electrodes, the locations and constellations of which are indicated by the letters in the schema of the electrode layout. ERPs shown here correspond to responses evoked by the first four repetitions, as well as the averaged responses that occurred in the intermediate and late repetition phases. Specific ERP-components are indicated for the middle parietal electrodes (J)

**Fig. 4:**
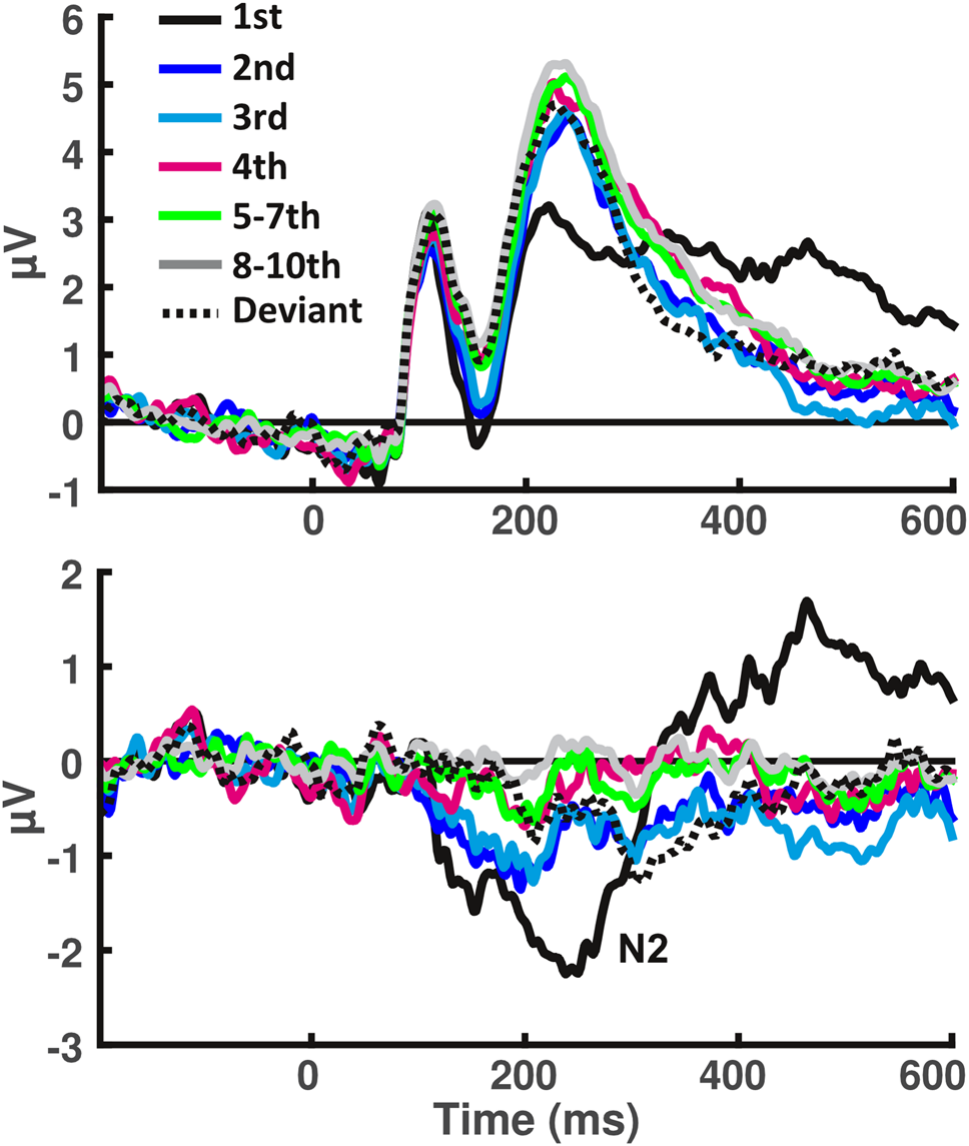
Human ERP and difference waves from the parietal electrode, showing N2 used for analysis. Top: original human grand average ERPs of all conditions in electrode P2. Bottom: difference waves of all conditions for electrode P2 in humans

Examination of the ERPs generated by the visual stimuli (Fig. 3a–n) identified different stages corresponding to different ERP-components (see top of Fig. 2, for parietal example), such as the P1 for primary visual areas, and the N1 for inferotemporal source power. The P2 corresponded to the parietal power increase during its upward slope, and the posterior cingulate activity during its descending phase, although this component appeared to be less characterized by any particular source estimation pattern. Furthermore, in this downward slope, we found that the novelty ERPs displayed a markedly more negative amplitude relative to repeated objects (Fig. 4). We hypothesized this to be the N2, and thus considered this an individual component that we then investigated based on each conditions’ difference amplitude to the tenth presentation. This yielded what indeed appeared to be a component largely specific to the first presentation of a new item, with an amplitude of − 3.41 µV (SD 3.06), relative to the late repetition phase. The late, mostly frontally distributed, increase in source power corresponded temporally to the P300. The details of which electrodes and time windows we used for each component in the following analyses can be found in Table 1.

**Table 1:** List of human ERP-components: Component-labels, labels of electrodes (according to the 10% extension of the international 10–20 system) and latencies used for analysis, as well as the average (Av.) amplitude and the nature of the repetition (Rep.) effect

Label	Electrodes	Latency (ms)	Av. amplitude (µV)	Rep. effect
P1	*PO7, PO3, POz, PO4, PO8, O1, Oz, O2*	110	6.91 (3.64)	Linear
N1	*P5, P3, P1, Pz, P2, P4, P6*	150	0.7 (2.95)	Linear
P2	*PO7, PO3, POz, PO4, PO8, O1, Oz, O2*	240	10.76 (4.95)	Quadratic
N2	*P2 *(difference wave)	270–290	− 0.27 (3.46)	Quadratic
P300	*CP3, CP1, CPz, CP2, CP4, Pz*	300–500	1.69 (1.54)	Quadratic

### Rodent novelty response passes from V1 to parietal, lateral visual, and retrosplenial areas

**Fig. 5:**
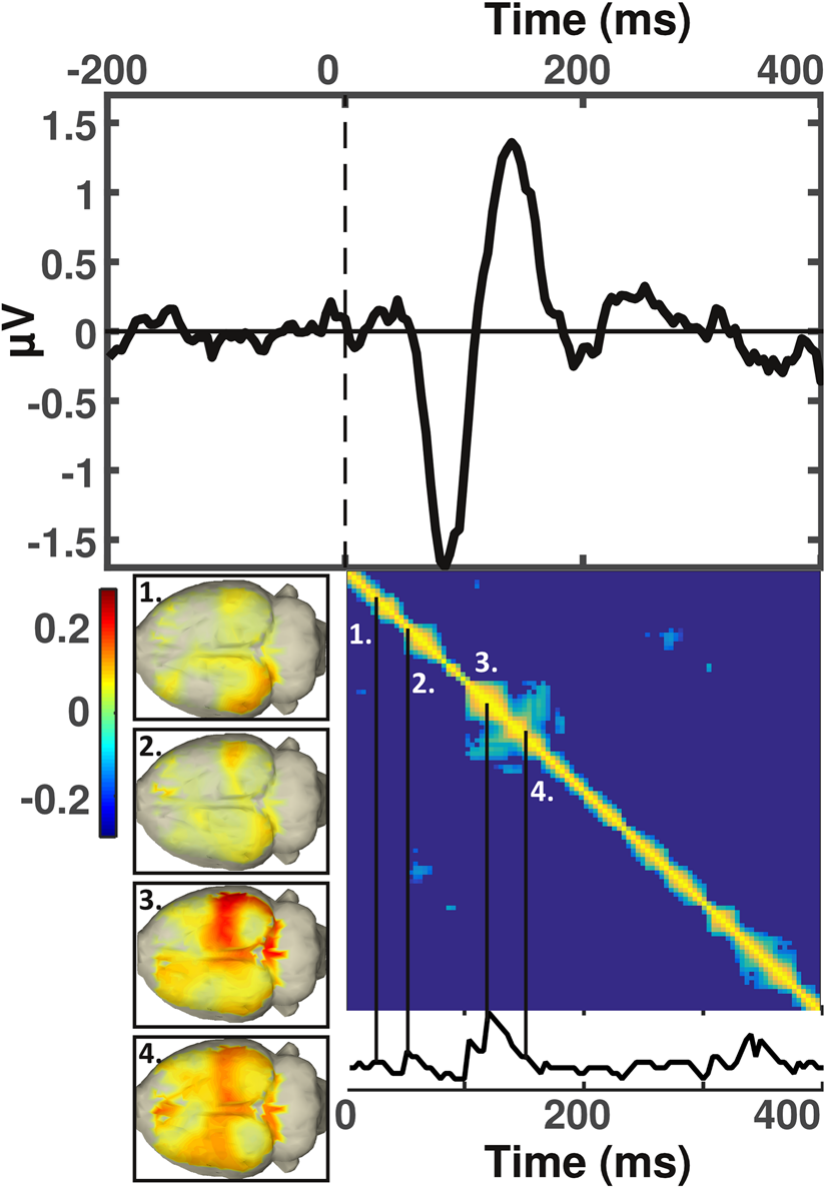
Rodent event-related potential sources elicited by visuospatial information processing. Top: rodent grand average event-related potential (ERP) derived from posterior electrodes (situated above V1). Bottom right: representational similarity matrix calculated for the time span of 0–400 ms and with the significance level at at *p* < 0.05 that includes a time series indicating across how many time points source-patterns correlated significantly. Bottom left: source estimates for time windows of interest corresponding to the numbering included in the bottom right panel

In the rodents, we found an initial activation of visual cortices at around 32–40 ms (Fig. 5, inset ‘1.’). This corresponds to the neuronal response latency in the primary visual cortex (Vermaercke et al. 2014). This then progressed to parietal areas starting from 52 ms (Fig. 5, inset ‘2.’), from where the novelty response simultaneously extended towards occipito-temporal areas at 120 ms (Fig. 5, inset ‘3.’), as well as to the retrosplenial cortex, and at 152 ms to the anterior cingulate region (Fig. 5, inset ‘4.’). Despite the brevity and short latencies of the early rodent ERP-components and the corresponding stages, the rodent brain patterns also displayed several peaks in temporal coherence, suggesting that the localization to the visual cortex appears to be temporally separable from the subsequent increase in parietal estimates (Fig. 5). The subsequent spread to occipito-temporal, retrosplenial, and finally also to anterior cingulate areas appeared to form a complex. We also identified a late temporal coherence in brain patterns, between 300 and 400 ms, which did not correspond to any particular cortical increases or decreases in source power (and hence formed its own discrete stage). Given that, at the electrode level, the novelty response was also prominent at this time point (see below), the lack of source differences may have arisen from a larger amount of variance over time in rodents, or be indicative of a subcortical generation mechanism.

### Rodent ERP-components reflect discrete stages of novelty processing

**Fig. 6:**
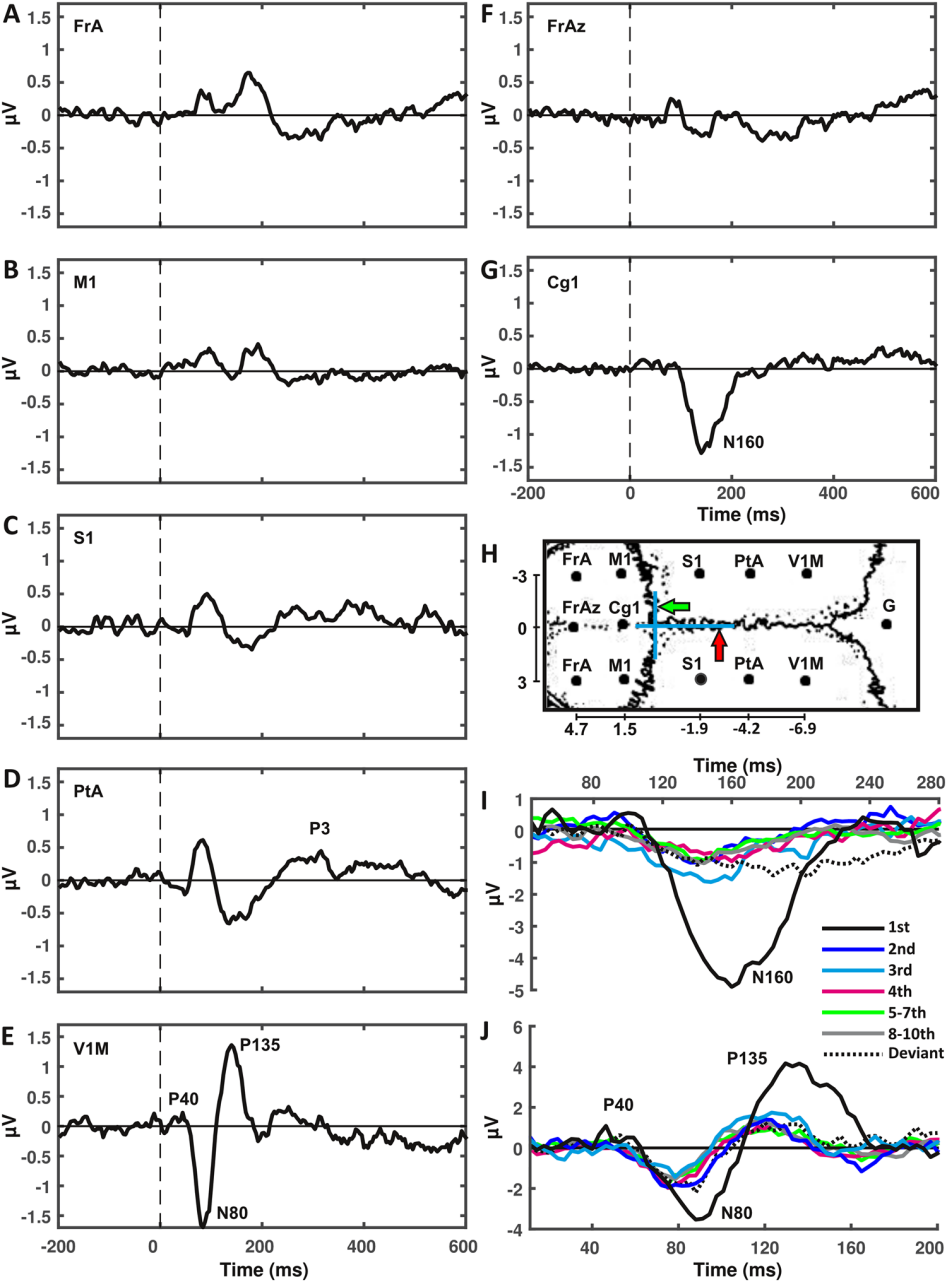
Rodent visual event-related potentials respond to novelty. **a**–**e** Rodent grand average ERPs averaged over lateral electrodes across all conditions are shown (note that for **c** we only display the left electrode); **f**–**g** grand average ERPs from the midline electrodes; **h** electrode layout. Green arrow indicates Bregma (overlaid by vertical blue line), red arrow indicates the midline (overlaid by horizontal blue line). *Cg1* cingulate cortex area 1, *FrA* frontal association area, *FrAz* central frontal association area, *G* ground, *M1* primary motor cortex, *PtA* parietal association area, *S1* primary somatosensory cortex, *V1M*: primary visual cortex, monocular area, *I* N160 in Cg1-electrode, *J* P40, N80, and P135 in occipital electrodes

The rodent ERPs (Fig. 6a–j) displayed a similar progression as that observed for the human potentials, in that a small positivity at 40 ms (P40) was observed in occipital electrodes. This corresponded from a temporal point of view to the stage indicating the increase in primary visual cortex source estimates. Following this, we found a negativity at 80 ms (N80), that was most prominent in occipital electrodes (Fig. 6e, j), and which corresponded to the second stage we identified in the parietal source power increase. Following the N80, we found a positive deflection, again in occipital electrodes, at roughly 135 ms (P135; Fig. 6e, j). During its descending phase this deflection overlapped with a negativity at 160 ms (N160; Fig. 6g, j) that was very pronounced in the second middle electrode, and was temporally in line with anterior cingulate activity. Together, the P135 and the N160 corresponded to the rapid distribution from parietal to temporo-occipital regions, the retrosplenial area, and the anterior cingulate. We also found an extended positivity in parietal electrodes from roughly 200 to 500 ms, that we tentatively labeled P3 (Fig. 6d), more due to its position within the ERP-component sequence, rather than as a suggestion of it being analogous to the human P300.

### Repetition effects in human subjects are mostly linear and the deviant response occurs during the N2

**Fig. 7:**
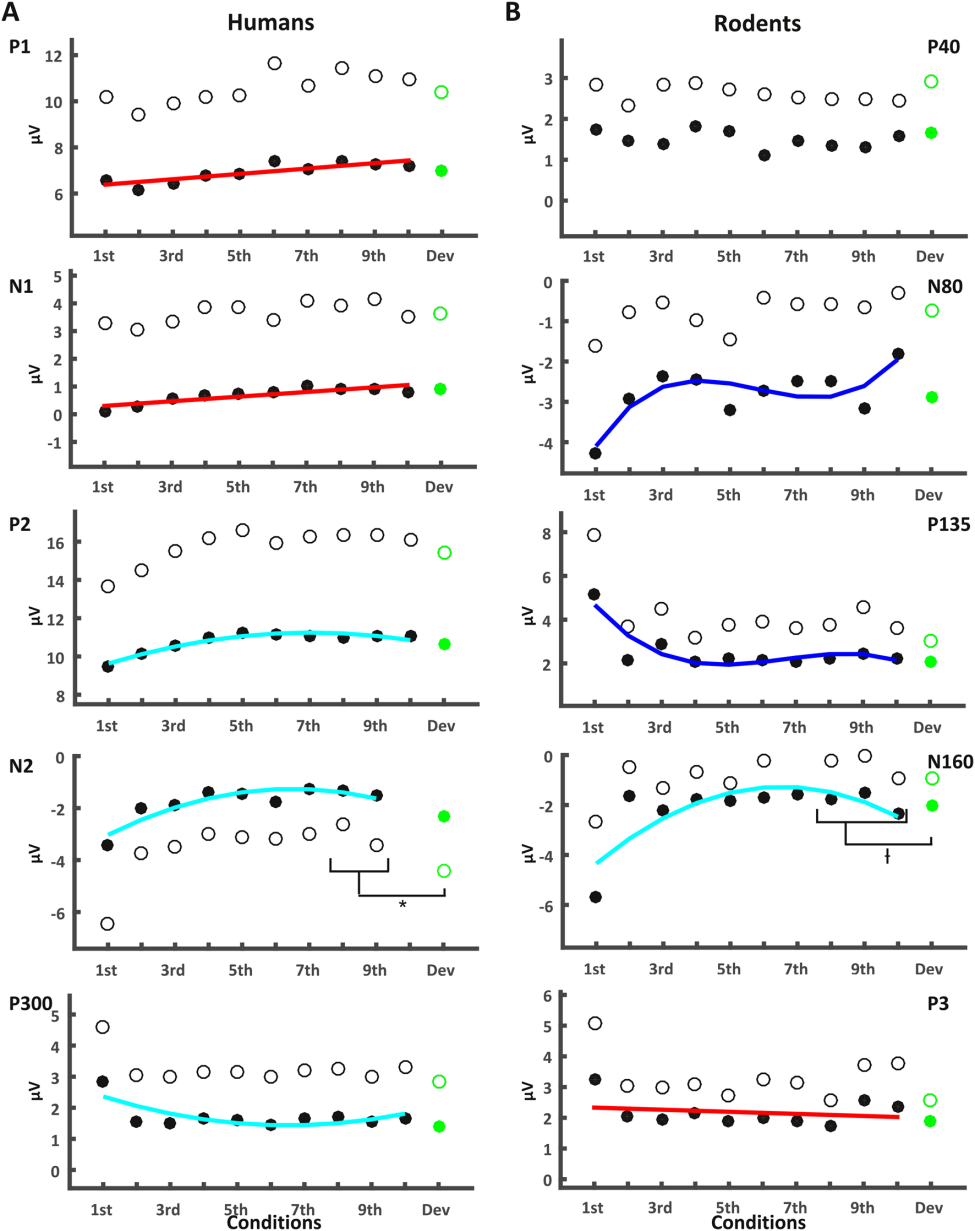
Human repetition effects are mostly linear, rodent effects are mostly non-linear. Amplitudes for the repetitions (black dots, empty dots indicating standard deviation) and the deviant (green) different ERP-components, arranged in sequence of their occurrence for humans (left) and rodents (right). Red, cyan, and blue lines correspond to the linear, quadratic, or cubic polynomial functions fitted to the subject-averaged amplitudes, respectively, depending on which function explained the effects best. An asterisk indicates a significant difference between the mean amplitude of the late repetition trials the deviant. A cross indicates a significant deviant effect for this component, corresponding to a slightly later time window

The P1 component (Fig. 7a) showed a significant repetition effect, *F*(5, 130) = 5.32, *p* = 0.001. The linear, quadratic and cubic functions applied to the amplitudes of each time point averaged over subjects yielded *R*^2^_adj_ of 0.66, 0.66, and 0.79, respectively. However, the Wilcoxon signed rank test did not indicate a significant difference when comparing these parameters derived from fitting each subjects time–amplitude data individually, *z* = − 0.192, *p* = 0.84, and *z* = 0.096, *p* = 0.923, for the comparisons of linear against quadratic and cubic fits, respectively. The effect of repetition was thus more of a linear nature. There was no significant effect of deviant stimuli on the P1 component, *t*(26) = − 1.02, *p* = 0.32.

For the N1 (Fig. 7a), we found a significant amplitude reduction over the repetition sequence, *F*(5, 130) = 5.45, *p* = 0.003. The linear function was not significantly worse than the quadratic or cubic functions at explaining the data with *R*^2^_adj_ = 0.691, 0.955 and 0.951, respectively, *z* = 0.985, *p* = 0.325, *z* = − 1.87, *p* = 0.061. Again, the deviant effect was found to be insignificant *t*(26) = 0.32, *p* = 0.74.

The P2 (Fig. 7a) was also found to display a significant effect of repetition, *F*(5, 130) = 9.06, *p* < 0.001, that was deemed to be following a quadratic function, with an average *R*^2^_adj_ = 0.498, 0.914, and 0.983 for the linear, quadratic, and cubic functions applied on the averaged data, and *z* = − 2.306, *p* = 0.021, *z* = − 1.393, *p* = 0.164 for the comparisons between linear, and quadratic or cubic fits, respectively. Based on our increased *α* level, the deviant effect was non-significant, *t*(26) = − 2.1, *p* = 0.045.

The N2 (Fig. 7a), for which we conducted all analyses omitting the tenth presentation that instead served as a baseline to produce the difference waves, also showed a significant effect of repetition, *F*(5, 130) = 10.49, *p* > 0.001. With an *R*^2^_adj_ = 0.454, 0.763, and 0.842, on the grand average, the quadratic function proved to be a better fit, compared to the linear function, with *z* = − 2.09, and *p* = 0.037, while the cubic function did not, *z* = − 1.345, *p* = 0.179. Furthermore, this difference wave showed a significant deviant effect, with the deviant condition producing a difference amplitude of − 2.32 (SD: 2.11), compared to 0.96 (SD 1.4) found as an average of the eighth and ninth standard presentations, *t*(26) = − 2.78, *p* = 0.01.

Finally, the P300 (Fig. 7a) showed a strong novelty response, which lead to a significant repetition effect, *F*(5, 130) = 20.54, *p* < 0.001. In contrast to all the previous components, this effect was found to be better explained by both the quadratic and cubic functions, rather than the linear function, with *z* = − 2.619 and *p* = 0.009 (for the quadratic comparison) and *z* = − 2.955 and *p* = 0.003 (for the cubic comparison). Comparing the quadratic and cubic functions among each other yielded no significant differences, *z* = − 1.634, *p* = 0.102. The respective goodness-of-fit on the subject averaged data was *R*^2^_adj_ = 0.107, 0.399, and 0.666 for the linear, quadratic, and cubic functions, respectively. The deviant effect did not reach our significance threshold, with *t* (26) = − 2.37, *p* = 0.025.

In summary, for the human components, we consistently found significant effects of repetition, that were linear up until the P2, where a quadratic polynomial function was a better fit, as was the case for the N2 and P300 components. Deviant effects, in turn, were only found in the N2 difference wave.

### Repetition effects in the rodent are mostly non-linear, while the N160 is sensitive to deviance

The rodent P40 (Fig. 7b) was not significantly altered by the repetition of the standard stimuli, or the presentation of a deviant, *F*(5, 60) = 0.41, *p* = 0.73, and *t*(12) = 0.67, *p* = 0.51, respectively.

The N80 in turn showed a significant main-effect of repetition, *F*(5, 60) = 3.85, *p* = 0.018 (Fig. 7b; Table 2). This effect showed a trend towards being better explained by the cubic function, with *z* = − 1.922, and *p* = 0.055, whereas the quadratic function did not yield a comparable decrease in error, *z* = 0.035, *p* = 0.972. The deviant effect was insignificant *t*(12) = − 1.12, *p* = 0.28.

**Table 2:** List of rodent ERP components: Their assigned labels, which electrodes and latencies were used for analysis, as well as their average amplitude and nature of the repetition effect, if present

Label	Electrodes	Latency (ms)	Av. amplitude (µV)	Repetition effect
P40	*V1M* (L. & R.)	40	2.42 (3.28)	NS
N80	*V1M* (L. & R.)	80	− 1.15 (3.26)	Cubic (trend)
P135	*V1M* (L. & R.)	134	2.9 (3.33)	Cubic
N160	*CG1*	270–290	− 0.85 (2.98)	Quadratic
P3	*PtA* (L. & R.)	300–400	2.63 (3.23)	Linear

The P135 showed a strong repetition effect, with *F*(5, 60) = 11.83, *p* < 0.001, that was best explained by the cubic function *z* = − 2.83, *p* = 0.005 (Fig. 7b). With *R*^2^_adj_ = 0.188, 0.514, and 0.657, for average data fitted with linear, quadratic, and cubic functions, respectively, the difference between the quadratic and linear goodness-of-fit was not significantly different, *z* = − 1.433, *p* = 0.152. No significant deviant effect was found, *t*(12) = − 0.4, *p* = 0.7.

The N160, that we found restricted to the anterior cingulate electrode (Fig. 6g), also displayed a very strong novelty response, in line with a significant effect of repetition, *F*(5, 60) = 17.05, *p* < 0.001 (Fig. 7b). Again this rodent component, with a linear fit of *R*^2^_adj_ = 0.156, a quadratic fit of *R*^2^_adj_ = 0.514 and a cubic fit of *R*^2^_adj_ = 0.592, was significantly non-linear over repeated presentation of standards, with *z* = − 2.062, *p* = 0.039 for the linear–quadratic comparison and *z* = − 2.201, *p* = 0.028 for the linear–cubic comparison. Quadratic and cubic functions did not differ from one another *z* = 1.013, *p* = 0.311, suggesting that this component was more aligned with the human components P2 or N2 in its behavior across repetitions. Furthermore, a variant of this component in the form of a slower and slightly later onset was found to be significantly responsive to deviants, based on a *t* test on its mean amplitude *t*(12) = − 3.92, *p* = 0.002.

Finally, as the parietal P3 (Fig. 6d) extended across approximately 140 ms from 220 to 360 ms, we used the largest positive amplitude in the two parietal electrodes during this time window for comparison. This component displayed a strong repetition effect, *F*(5, 60) = 6.26, *p* < 0.001 (Fig. 7b). However, despite progressing very similarly to the human P300, this component turned out to be not significantly better explained by the non-linear function, which produced a fit of *R*^2^_adj_ = 0.488 (for the quadratic function) and *R*^2^_adj_ = 0.496 (for the cubic function), as opposed to the linear function that in fact displayed a negative *R*^2^_adj_ of − 0.066, when fitting these curves to the subject average data points. When comparing the fits of linear and the two non-linear functions across subjects, the Wilcoxon test reported *z* = − 1.153, *p* = 0.249, and *z* = − 0.384, *p* = 0.7 for the comparison between linear and quadratic/cubic functions. Furthermore, the deviant effect was not significant, *t*(12) = − 1.11, *p* = 0.29. This may in part be due to the recovery of amplitudes in the ERPs of the ninth and tenth presentations in this component, or the aforementioned variance across our rodent cohort, both in terms of neural generators for the P3, as well as time.

To summarize, the early rodent components that were sensitive to repetition responded in accordance with a cubic polynomial function, whereas the human cohort displayed almost the opposite behavior, in that all components up until the P300 responded according to a linear or quadratic function. One striking commonality, however, is that both species responded to deviants in a late negativity (the rodent N160, and human N2) that coincided with the descending phase of the second positive deflection (the rodent P135 and human P2). The source estimates for this time window in both species appeared to involve cingulate, and potentially retrosplenial, areas.

## Discussion

The present study compared the cortical dynamics involved in object perception in rats and humans. This was done by engaging both species in an equivalent visuospatial task that involved showing them images of abstract, yet real, objects that were selected to minimize affordance characteristics. Both species viewed the images in an oddball-like paradigm, where objects were repeated up to 21 times, including one subtle configurational deviant, before a different object was shown in the same manner.

We found that source estimation of the novelty response in humans conforms with the hypothesized activation of ventral pathway regions, such as the inferotemporal cortex, that is central to object perception. This corresponded to the N1 component, that has, in previous studies, been linked to discrimination tasks (Vogel and Luck 2000; Hopf et al. 2002), although usually dependent on attention. Perhaps the most pronounced effect identified by our source estimates was the strong increase in the area of the posterior midline, near the cingulate, or retrosplenial areas, in the time window of the P2 and N2. Interestingly, this was also the only component sensitive to the deviant stimulus.

The source estimates of the rodents displayed a comparable progression, in that visual cortex activity at around 40 ms was followed by a very rapid spread of increased source power from the parietal to lateral occipital and retrosplenial areas, from 80 ms to roughly 150 ms after stimulus presentation. The eccentricities that we found in the ERPs corresponded well to the stages that were separable from one another in the multivariate correlation analysis. More importantly, together with the source estimates, the first ERP-components appear to fit neatly to those identified using single-unit recordings by Vermaercke et al. (2014). They reported neuronal responses in visual cortices at a latency of 40 ms, followed by lateral areas at around 80 ms. This would suggest that the spread from primary to lateral visual areas is indeed representative of complex object processing. Taking into account an overall shorter response latency in the rodent, this may correspond to the occipital and inferior temporal increase in source power observed during the P1 and N1 in humans. One difference in the progression of novelty responses that we identified in rodents and humans is that in the rat, the parietal area became involved in novelty processing at an earlier stage than seen in humans. This contrasts to reports from primate studies that describe that the parietal cortex, as a part of the dorsal stream, fulfills an action-centered role (Goodale et al. 1991; Goodale and Milner 1992).

We would like to speculate that the rodent P135 and N160 correspond to the human P2 and N2. However, in our human subjects, the P2 and N2 were difficult to disambiguate on a temporal and topographical level, and the rodent P135 and N160 also appeared to form a complex. Thus this question remains unanswered. Nonetheless, both the human P2/N2 and the rodent P135/N160 were responsive to deviants, and appeared to both involve posterior cingulate, or retrosplenial, areas. Possibly these complexes of the N2/P2 and P135/N160 may reflect a higher order mismatch detection that may be comparable between the species (Czigler et al. 2006; Winkler and Czigler 2012; Stefanics et al. 2014).

How do the early rodent ERP-components we observed in our visuospatial paradigm compare to previous studies of rodent ERPs? Most other studies, especially those that targeted more complex processing streams in the context of comparative investigations of human and rodent ERPs, have largely been restricted to the auditory modality (e.g., Brankačk et al. 1996; Ehlers et al. 1994; Hurlbut 1987; Meeren et al. 2001; Nguyen and Lin 2014; Sambeth and Maes 2006; Sambeth et al. 2003, 2004). Some studies have identified a number of different components that appear within roughly 50 ms after stimulus onset (de Bruin et al. 2001; Quian Quiroga and Van Luijtelaar 2002). Other studies reported components that extended over a longer period of 200 ms after stimulus onset (Sambeth et al. 2004; Sambeth and Maes 2006). Some of these components have been scrutinized in light of stimulus repetition. In a study that directly compared rats and humans, Sambeth et al. (2004) presented task-irrelevant tones and found that both species tended to have a similar progression of components, albeit at different latencies. These were labeled as P30, N70, P115, and N195, and considered as potential analogs of the human P1, N1, P2, and N2, respectively. A cross-species commonality of showing repetition effects was found for the N70/N1, the P115/P2, and the N195/N2, although the magnitude of those effects was different across species (Sambeth et al. 2004).

Finally, the question as to why both of our cohorts failed to show the expected deviance effect at the level of the P300/P3 remains. Given that in humans, the P300 is believed to reflect updating processes (Polich 2007), one potential explanation is that for our human cohort the deviant was too subtle to initiate this response. While this is purely speculative, we may still ask whether the rodent P3 is a candidate for a visually evoked P300. For the novelty response, the human P300 appeared to be mostly frontally distributed, whereas the rodent equivalent did not localize to a particular source, which may be due to the fact that it may arise from subcortical structures (Knight 1996). Furthermore, the human P300 and the rodent P3 were characterized by different electrophysiological profiles, although overall, their progressions over repetitions appeared to be almost identical (Fig. 7). However, given that neither human nor rodent showed the classical P300-response to deviant stimuli warrants caution with regard to such interpretations.

A component potentially equivalent to the human P300 has repeatedly been reported in active auditory oddball paradigms performed by rats, where the animals were trained to respond to a particular deviant tone (Hurlbut 1987; Yamaguchi et al. 1993; Ehlers et al. 1994; Brankačk et al. 1996; Shinba 1997; Sambeth et al. 2003). These findings have been ambiguous with regard to the latency of this component, with some studies suggesting a proportionate latency to the preceding components, thus an earlier occurrence than in humans (Yamaguchi et al. 1993), and others reporting a more variable, potentially even later occurrence of the rodent P3 relative to the human equivalent (Sambeth et al. 2003). This may also have been a factor in the current study, in that there may be more variability in how and when the rodent P300-equivalent is elicited.

In conclusion, this study offers the first description and comparison of the broad topography of ERPs recorded from rats and humans during passive perception of complex and abstract objects. We found a similar progression of the novelty response in both species, whereas the response to object repetition differed between species. Interestingly, both species responded to deviant configurations of the objects in a similar way. These results suggest the ERPs evoked by passive visuospatial information processes exhibit a surprising degree of equivalency despite the vastly different cognitive capacities of the species studied. These findings suggest that ERPs generated in rats by novelty and deviance in this manner could serve as a basis for the study of the underlying neural, synaptic, and neurobiological mechanisms of the equivalent response in humans.
